# *Drosophila* tools and assays for the study of human diseases

**DOI:** 10.1242/dmm.023762

**Published:** 2016-03-01

**Authors:** Berrak Ugur, Kuchuan Chen, Hugo J. Bellen

**Affiliations:** 1 Program in Developmental Biology, Baylor College of Medicine, Houston, TX 77030, USA; 2 Department of Molecular and Human Genetics, Baylor College of Medicine, Houston, TX 77030, USA; 3 Department of Neuroscience, Baylor College of Medicine, Houston, TX 77030, USA; 4 Howard Hughes Medical Institute, Baylor College of Medicine, Houston, TX 77030, USA; 5 Jan and Dan Duncan Neurological Research Institute, Texas Children's Hospital, Houston, TX 77030, USA

**Keywords:** *Drosophila*, Human disease models, Nervous system, Neurodegeneration, Regeneration, Heart, Liver, Oenocyte, Fat body, Kidney, Nephrocytes, Malpighian tubules

## Abstract

Many of the internal organ systems of *Drosophila melanogaster* are functionally analogous to those in vertebrates, including humans. Although humans and flies differ greatly in terms of their gross morphological and cellular features, many of the molecular mechanisms that govern development and drive cellular and physiological processes are conserved between both organisms. The morphological differences are deceiving and have led researchers to undervalue the study of invertebrate organs in unraveling pathogenic mechanisms of diseases. In this review and accompanying poster, we highlight the physiological and molecular parallels between fly and human organs that validate the use of *Drosophila* to study the molecular pathogenesis underlying human diseases. We discuss assays that have been developed in flies to study the function of specific genes in the central nervous system, heart, liver and kidney, and provide examples of the use of these assays to address questions related to human diseases. These assays provide us with simple yet powerful tools to study the pathogenic mechanisms associated with human disease-causing genes.

## Abstract (precis)

**Editors' choice - *Drosophila* Collection:** In this review and accompanying poster, we highlight the physiological and molecular parallels between fly and human organs that validate the use of *Drosophila* to study the molecular pathogenesis underlying human diseases.

## Introduction

The fruit fly has come a long way since Charles W. Woodworth, an American entomologist, first proposed to use *Drosophila melanogaster* as a genetic model organism in 1900 (Sturtevant, 1959). In the past 100 years, fly research has been particularly valuable for the analysis of molecular mechanisms underlying genetic phenomena, behavior and development. Approximately 65% of human disease-causing genes are believed to have a functional homolog in flies (Chien et al., 2002; Yamamoto et al., 2014) and a significant fraction of these homologs are expressed in *Drosophila* tissues that perform the function of the equivalent human tissue (Chintapalli et al., 2007). In our opinion, the evolutionary conservation of genes and their associated functions has not yet been exploited to its full potential, particularly in translational biology. Given the genetic tractability of flies and the many tools available for their genetic manipulation, numerous studies can now be performed in flies to more rapidly discover the molecular mechanisms by which human mutations cause disease phenotypes.

**Figure:**
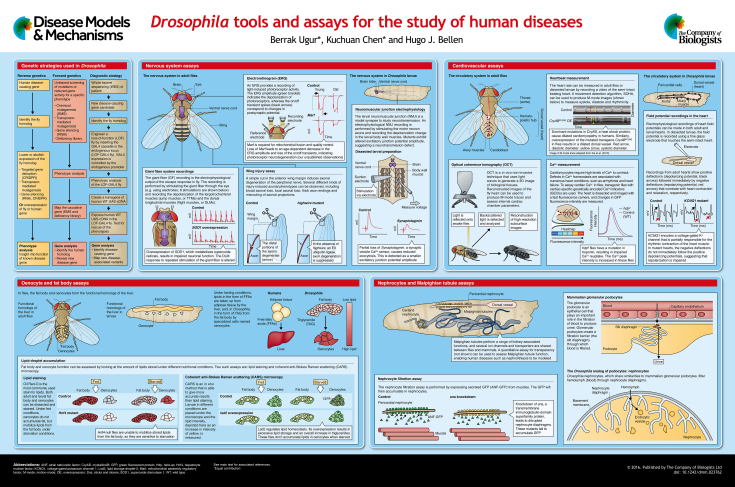

Broadly speaking, three main strategies to study human diseases using fly models have been developed: (1) reverse genetics, (2) forward genetics and (3) a recently established strategy to aid in the discovery of human disease-causing genes, which we name ‘diagnostic strategy’ (see the first panel in the poster). In the reverse genetics approach, mutations are created in fly homologs of human genes to study their phenotypes *in vivo*. There are mainly three ways to lower or abolish expression of a gene in flies; targeted gene disruption [e.g. using clustered regularly interspaced short palindromic repeats/Cas9 (CRISPR/Cas9)] (Beumer and Carroll, 2014), transposon-mediated mutagenesis and excision of existing transposable elements (TE), and gene silencing [via RNA interference (RNAi), CRISPR] (Mohr, 2014). In addition to loss-of-function studies, a wild-type or mutant version of a human disease-causing gene (transgene) can be overexpressed in flies to assess the effects in specific tissues (Feany and Bender, 2000).

In forward genetics, mutations are induced at random and the animals are screened for a particular phenotype. Mutations can be generated chemically [e.g. using ethyl methanesulfonate (EMS)] or by using transposons (Venken and Bellen, 2014), or mutants can be isolated by screening an RNAi library or a collection of existing deficiencies (Cook et al., 2012). This is an unbiased strategy that can help identify uncharacterized mutations in known disease genes (phenotypic expansion) as well as genes that have not been previously linked to disease. Forward genetics can thus be a powerful driving force for identifying previously unknown genes and unraveling biological phenomena.

Lastly, through the diagnostic strategy, *Drosophila* can be used to assess the pathogenic properties of rare variants that have been linked to human diseases. There are many United States (USA)-based initiatives designed to identify human disease-causing genes by sequencing the whole exome or genome of patients and their siblings or parents, coordinated by e.g. Centers for Mendelian Genomics (http://www.mendelian.org/) and the Undiagnosed Diseases Network (UDN; http://undiagnosed.hms.harvard.edu/). Similar strategies have been adopted in other countries, including the UK (http://www.uk10k.org/), China (Guangzhou *Drosophila* Resource Center and the Center for Genomic Sciences in the University of Hong Kong), and many more. This strategy is often inadequate to determine the causative gene variant when three or fewer individuals are assessed. We developed a pipeline that overcomes this challenge and enables the causative mutation to be pinpointed. First, the fly homolog or ortholog is knocked out by integrating a *GAL4* gene under the control of the endogenous regulatory elements, and the phenotype is assessed. If this phenotype can be rescued by expression of the wild-type *UAS*–human-cDNA but not by the human variant, causality is determined (Bellen and Yamamoto, 2015; Wangler et al., 2015).

In the following sections we will describe assays designed for key *Drosophila* organ systems that permit the study of homologs of human genes that cause disorders affecting the nervous system, heart, liver and kidney. We refer readers to other reviews focused on the fruit fly tracheal system (Wagner et al., 2008), peripheral nervous system (Charng et al., 2014) and gut (Apidianakis and Rahme, 2011), because these organ systems are not discussed here.

## Nervous system assays

The nervous system in *Drosophila* is required for sensing and processing information related to vision, hearing, olfaction, proprioception and taste. Just like in humans, this information is conveyed to the CNS and processed to provide a motor output. Although the gross anatomy of *Drosophila* and human brain is very different, they share numerous conserved genetic, cellular, electrophysiological and chemical properties. As in vertebrates, many different types of neurons are required to process information in fruit flies. For example, in the visual system, ∼115 different types of neurons have been identified in *Drosophila*, which is a very similar number to what has been estimated in vertebrates (Venken et al., 2011). However, there are probably a million-fold fewer neurons overall in flies than in vertebrates. The reduced complexity and ease to study the *Drosophila* nervous system allows an in-depth assessment of the function of genes and neuronal networks. Many different assays have been developed to assess neuronal function. These include hearing, flight, learning and memory, and diurnal rhythmicity assays, as well as numerous highly specific behavioral assays (Branson et al., 2009; Inagaki et al., 2010; McGuire et al., 2005; Simon and Dickinson, 2010). In the last two decades, *Drosophila* has been increasingly used to model neurological dysfunction, including neurodegeneration, epilepsy, dementias, stroke, traumatic brain injury and brain tumors. In this section, we provide a snapshot of the different assays that can be used to study neurological disorders and describe the specific contexts in which they have been most useful so far. These tools allow researchers to gain novel insights into pathogenic mechanisms and might help to provide new therapeutic strategies for different neurological diseases.

### Electroretinogram

Unlike vertebrate photoreceptors (PRs), which are light-sensory cells in the retina that connect to a neuron, fly PRs themselves are light-sensory neurons that project axons into the deep lamina and medulla layers of the adult brain. The ease of accessibility of fly PRs and their regular arrangement have facilitated the development of several assays to study retinal disorders and other more general neurological phenotypes. The electroretinogram (ERG) records the change in electrical activity of the PRs in response to a flash of light. The ERG depolarization amplitude provides a read-out of the phototransduction process, whereas the on/off transient spikes at the onset and offset of a light flash correspond to postsynaptic potential changes (see poster) (Hardie and Raghu, 2001; Stark and Wasserman, 1972).

ERG recordings were adapted to assess mutant phenotypes for *Drosophila* in the late 1960s (Hotta and Benzer, 1969; Pak et al., 1969). Given the ease of the assay, forward genetic screens based on ERGs in homozygous viable mutants permitted the identification of numerous genes that control the phototransduction pathway (Wang and Montell, 2007). Impaired phototransduction results in aberrant ERG recordings; for example, reduced depolarization amplitude and/or loss of the on and off transients (see example in the poster). Most of the mutations that affect the phototransduction pathway do not affect viability because the eye is not an essential organ in the fly. To circumvent the issue of lethality and study the role of essential genes in the eye, a technique was developed to generate eye-specific mosaic clones (Newsome et al., 2000; Stowers and Schwarz, 1999). This allowed the identification of genes that are more broadly implicated in neurodegeneration by measuring ERGs in young and old animals to document the time-dependent change in neuronal function (Haelterman et al., 2014; Yamamoto et al., 2014). Using this approach, the Bellen lab identified several fly mutants in which the human homologs have been implicated by other studies to play a role in various neurodegenerative diseases, including Leigh syndrome (*C8orf38/sicily*) (Zhang et al., 2013b), Charcot-Marie-Tooth disease type 2A (*MFN2/Marf*) (Sandoval et al., 2014) and autosomal recessive spastic ataxia with leukoencephalopathy (*MARS2*/*Aats-met*) (Bayat et al., 2012). In addition, we recently determined that reactive oxygen species (ROS) accumulate in the PRs of these mutants and, in turn, this triggers lipid-droplet formation that eventually contributes to PR neurodegeneration (Liu et al., 2015). These examples illustrate how ERGs can be effective tools to identify genes and elucidate molecular mechanisms underlying neurodegeneration.

### Neuromuscular junction electrophysiology

The neuromuscular junction (NMJ) is the connection (synapse) between the motor neuron and the muscle. Disorders of the NMJ span a variety of phenotypes and can be due to genetic or acquired causes (Rodríguez Cruz et al., 2014). The *Drosophila* NMJ provides a powerful platform to study neuromuscular diseases because it allows detailed analyses of structural connections between the neuron and the muscle as well as their electrophysiological properties. The fly larval NMJ consists of arrays of overlapping striated muscle fibers that are innervated by motor neurons that form synaptic boutons (Prokop and Meinertzhagen, 2006). The fly NMJ is a large glutamatergic synapse that is easy to access, thereby permitting a detailed characterization of the properties of synaptic transmission, including assessment of excitatory junctional potentials (EJPs), spontaneous miniature EJPs, synaptic plasticity, transmission electron microscopy imaging and pre- versus postsynaptic phenotypic analysis. Below, we discuss the NMJ electrophysiological assay (see poster).

The electrophysiological recording assay for the third instar larval NMJ was described in the seventies (Jan and Jan, 1976), and a parallel assay based on patch clamping in embryos was developed later (Broadie and Bate, 1993). Briefly, the larva or embryo is filleted to expose the muscles along the body wall. A motor neuron is cut posterior to the ventral ganglion and drawn into an electrode in order to induce action potentials. A second electrode is placed in or on the muscle to measure the response. Both assays permit a very accurate characterization of a mutant's neuronal function but they are quite labor-intensive and thus are not the best tools to perform genetic screens. Instead, genetic screens for altered NMJ morphology have been performed to identify genes regulating synaptic bouton morphology (Aberle et al., 2002) because the reiterated pattern of NMJs along the body wall lends itself to rapid visual inspection. NMJ electrophysiological assays have allowed detailed characterization of numerous mutant genes whose human homologs were later shown to cause different human diseases. For example, *Shaker* (*Sh*) and *ether-a-go-go* (*eag*) mutations were isolated owing to the shaking phenotype of the legs (Kaplan and Trout, 1969). Subsequently, further characterization of these mutants using NMJ electrophysiological recordings revealed that the mutations affect potassium channel function (Jan et al., 1977; Wu et al., 1983). The vertebrate homologs of these potassium channels were later identified and shown to be involved in many human diseases, including cardiac arrhythmias, deafness and epilepsy (Jentsch, 2000). Another example is the *Drosophila* homolog of the human genes synaptotagmin 1 and 2, which was first shown in flies to be the calcium (Ca^2+^) sensor for synaptic transmission, based on altered NMJ recordings (see poster) (Littleton et al., 1994). Recently, mutations in human synaptotagmin 2 have been discovered to cause Lambert-Eaton myasthenic syndrome, a rare autoimmune neuromuscular disorder (Herrmann et al., 2014).

Of note, the properties of *Drosophila* larval NMJs can be further studied using a variety of different assays, allowing an in-depth analysis that is not possible at any other synapse. These include live imaging of protein or organelle trafficking in the motor neuron axon or synapse (Andlauer and Sigrist, 2012), Ca^2+^ imaging of synaptic boutons (Macleod, 2012) and focal patch recordings from single boutons (Kurdyak et al., 1994). Given that fly NMJs are glutamatergic in nature, the similarities to mammalian CNS synapses provide us with a tool to study pathogenic mechanisms associated with neurological diseases, such as amyotrophic lateral sclerosis (ALS), spinal muscular atrophy and certain encephalopathies (Nahm et al., 2010; Pennetta et al., 2002; Sandoval et al., 2014; Sherwood et al., 2004; West et al., 2015). Collectively, these examples portray the instructive role of fly NMJ studies in identifying the pathogenic mechanisms of neuronal disorders.

### Giant-fiber-system recordings

Although NMJ electrophysiological assays in flies are very useful to study synaptic development and transmission, they do not enable the analysis of neuronal function over time. Adult-specific assays are more appropriate to document gradual changes that occur in neuronal function with age, and are thus particularly useful for the study of neurodegenerative disorders. The giant fiber system (GFS) is one of the few neuronal circuits in adult flies that is amenable to electrophysiological recordings (Tanouye and Wyman, 1980). When flies detect a change in luminescence, the giant-fiber neurons in the brain signal to the thoracic ganglion to activate flight muscles [dorsal longitudinal muscles (DLMs)] and jump muscles [tergotrochanteral muscles (TTMs)] (King and Wyman, 1980). Electrophysiological recordings of the GFS can be made by stimulating the eyes while recording the depolarization in DLMs and TTMs (Tanouye and Wyman, 1980) (see poster).

The GFS assay has been extensively used to study features of epilepsy and seizures by high-frequency stimulation in the eye and detection of seizure-like electrical activity in the muscles. For example, gain-of-function mutations in the fly gene *paralytic* (*para^bss^*) (Parker et al., 2011; Pavlidis and Tanouye, 1995) and loss-of-function alleles of *easily shocked* (Pavlidis et al., 1994) have a much lower threshold voltage of high-frequency stimulation to evoke seizure-like activity (Pavlidis and Tanouye, 1995)*. para* encodes a voltage-gated sodium channel (Feng et al., 1995), and its human homologs are either associated with or shown to cause diseases such as encephalomyopathy, neuropathy and myoclonic epilepsy (Escayg et al., 2000; Meisler and Kearney, 2005). Recently, GFS assays have been used to document the demise of neurons in neurodegenerative studies and provide insight into underlying pathologies (Kerr et al., 2011; Lee et al., 2004; Watson et al., 2008; Dutta et al., 2016). For example, mutations in superoxide dismutase 1 (SOD1) in humans cause ALS (Rosen, 1993). Flies that overexpress wild-type human *SOD1* are unable to follow high-frequency stimulation in DLMs but exhibit a normal TTM response, indicative of interference of the GFS (see poster). Flies that overexpress human mutant *SOD1* have defective TTM and DLM responses, which worsen with age, consistent with age-dependent degeneration in ALS (Watson et al., 2008). This fly ALS model demonstrates the convenience of the GFS in neurodegeneration studies because it detects the progressive decline in motor function through electrophysiology.

### Wing injury assay

The recently developed *Drosophila* wing injury assay is an elegant approach to study axonal degeneration and regeneration *in vivo* (Fang et al., 2012). The goal of these studies is to identify genes that are required for axonal degeneration and regeneration, and to identify the regulatory processes that are involved in spinal cord and nerve injuries. The fly peripheral nervous system has been intensively studied, which provides a great platform for this purpose. In the fly wing, mechanosensory and chemosensory neurons reside in the wing margin and project their axons toward the thoracic ganglion. Severing these axons using scissors or lasers causes degeneration of the distal portions of the axons (see poster) (Fang et al., 2012). After ∼7 days, the proximal portion of the injured axons regenerates by extending sprouts toward the lesion site. Alternatively, the axons regrow but invade another wing vein (Soares et al., 2014). These processes can be visualized by expressing a cytoplasmic green fluorescent protein (GFP) marker under the control of a neuronal *GAL4* driver. This simple assay is suitable for a large-scale forward genetic screen of viable as well as lethal mutations to identify genes that have not previously been implicated in degeneration or regeneration processes, because many of the genes and proteins required for these processes are evolutionarily conserved. By using this assay, a forward genetic screen identified mutations in *highwire*, a RING domain E3 ubiquitin ligase, that suppresses the degeneration of the distal portion of the axons upon axotomy (Neukomm et al., 2014). Furthermore, another screen based on this wing injury assay led to the discovery that downregulation of JNK signaling promotes axon regeneration (Soares et al., 2014). In summary, this novel technique capitalizes on the well-characterized fly peripheral nervous system and can provide clues about the molecular mechanisms that underlie the degeneration and regeneration of neurons.

## Cardiovascular assays

Fly models of cardiovascular diseases first emerged 20 years ago together with the development of assays to measure heart development and function (Bodmer, 1993; Ocorr et al., 2014). The *Drosophila* heart, called the dorsal vessel, differs from the human heart in that it is an open circulatory system consisting of a hollow, muscular tube closed at the posterior end. The vessel runs longitudinally from the posterior abdomen (heart proper) into the thorax (aorta). Similar to the human heart, which consists of distinct chambers, the fly heart is also divided into four chambers that are separated by small valve-like openings through which blood, or rather the analogous fluid in insects, hemolymph, enters the heart (Lehmacher et al., 2012). Each chamber consists of six myocardial cells to facilitate the flow of hemolymph through the dorsal vessel. The aorta, which is made up of myocardial cells that do not contract very much, is a tube that facilitates the transport of hemolymph to the head, from where it flows into the body cavity (Bier and Bodmer, 2004). The molecular pathways underlying the development of the fly heart and its function have provided valuable information relevant to human heart development and physiology. For example, *tinman *(*Nkx2-5* in humans), a homeobox transcription factor identified in flies, is required for heart development (Bodmer, 1993). Mutations in the human homolog of this gene were later shown to cause congenital heart disease and have subsequently been shown to cause sudden cardiac arrest in middle age (Schott et al., 1998). Moreover, the discovery of *pannier* (GATA4) and *neuromancer* (Tbx20) transcription factors revealed a conserved cardiogenic network, which enabled the study of these factors in human heart development and function (reviewed in Qian and Bodmer, 2012). The fly heart proves to be a convenient invertebrate heart disease model owing to conserved molecular pathways and the variety of assays to study different aspects of heart disease. In the following section, we will discuss a few of the cardiovascular assays and how they provide mechanistic insight about human heart disease. We refer readers to excellent reviews on different aspects of the topic for further information (Diop and Bodmer, 2015; Choma et al., 2011; Ocorr et al., 2014; Wolf and Rockman, 2011).

### Heartbeat measurement

Similar to mammals, the fly heartbeat consists of a cardiac cycle that includes diastolic and systolic periods. Interestingly, the cardiac cycle in adult flies is composed of alternating anterograde and retrograde beats (Wasserthal, 2007), leading to a periodic change in the flow of hemolymph (Dulcis and Levine, 2005). Measuring the heartbeat rate and rhythmicity is one of the fundamental assays to determine heart function. Measurements of the heartbeat, either in the dissected dorsal vessel of the larva or the adult abdomen, are based on visual recordings. These methods rely on the optical intensity of light passing through the heart while it beats (Gu and Singh, 1995). The approaches are relatively fast and can be used to assess the function of individual genes while also being appropriate for forward genetic screens.

Nowadays, further insight into cardiac function can be obtained by combining the standard heartbeat measurement assay with the use of a high-resolution camera and computer algorithms to detect optical intensity changes. This improvement of the method, semi-automated optical heartbeat analysis (SOHA), allows simultaneous assessment of systole, diastole and rhythmicity (Fink et al., 2009; Ocorr et al., 2009). This assay has been instructive in the study of various disease models, including channelopathies (Ocorr et al., 2007b), cardiomyopathies, age-dependent heart defects (Gill et al., 2015) and heart dysfunction associated with non-cardiac conditions, such as myotonic dystrophy (Chakraborty et al., 2015). Young wild-type flies monitored with SOHA show rhythmic cardiac contraction that gives rise to a characteristic M (motion)-mode trace. As these animals age, the contraction becomes arrhythmic (Ocorr et al., 2007a), analogous to cardiac arrhythmias observed in elderly humans (Jones, 2006). Apart from aging, the genetic cardiac fly models also recapitulate human cardiac disorders and allow in-depth analysis of genes involved in cardiovascular disease. For instance, dominant mutations in the human gene alpha-B crystallin (*CryAB^R120G^*), which encodes a chaperone, cause defects that underlie a range of diseases, including cardiomyopathies. Similarly, overexpression of the human *CryAB^R120G^* mutant transgene in the fly results in dilation of the dorsal vessel (see poster), mimicking dilated cardiomyopathy in humans (Xie et al., 2013).

### Optical coherence tomography (OCT)

The heartbeat assays described above all require the dissection of a semi-intact fly heart. More sophisticated tools such as optical coherence tomography (OCT), the equivalent of echocardiography in humans, permit the non-invasive characterization of the *Drosophila* heartbeat *in vivo* (Wolf et al., 2006). OCT uses laser beams to scan the entire tissue and subsequently uses the scattered light to produce subsurface images (see poster). This technique allows imaging of the heart in awake adult flies. This method is also convenient to produce M-mode images that reveal subtle changes in cardiac movement, similar to other video-based techniques. Yet, it should be kept in mind that, because this method requires downstream processing, it is relatively slow and is not very convenient to measure heart rhythmicity. Although it is costly and specialized, once established, it can be used for screening, however. Indeed, a deletion screen designed to identify cardiomyopathy genes via OCT led to the discovery that a component of Notch signaling, *weary (wry)*, could play a role in dilated cardiomyopathy (Kim et al., 2010). The protein encoded by *wry* has non-canonical Notch motifs, and overexpression of Serrate, a ligand of Notch, can rescue the cardiomyopathy in *wry* mutants. The results of this study indicate a potential role of Notch signaling in the adult heart and suggest that it can be a therapeutic target for dilated cardiomyopathies. In humans, Notch signaling is important for the organogenesis of the heart (de la Pompa and Epstein, 2012) and mutations in NOTCH1 cause aortic valve disease (Garg et al., 2005). Although the importance of Notch signaling in mammalian heart development has been studied, its role in the adult heart, especially in a regenerative context, is an emerging area that has many unexplored questions (Ferrari and Rizzo, 2014).

### Ca^2+^ measurement

Normal cardiac physiology requires controlled Ca^2+^ handling for proper contraction of cardiomyocytes, in flies as well as in humans. Defects in cardiac Ca^2+^ homeostasis are observed in cardiomyopathies and heart failures (Guo et al., 2006). Intracellular Ca^2+^ levels can be quantified using genetically encoded Ca^2+^ indicators (GECIs), such as GCaMP (a fusion protein of green fluorescent protein, calmodulin and a peptide sequence from myosin light chain kinase). These Ca^2+^ sensors have been successfully used to assess cardiac Ca^2+^ pulses by expressing them specifically in the fly heart (see poster) (Lin et al., 2011). Dilated cardiomyopathies are associated with altered Ca^2+^ homeostasis. For example, *held-up* mutant flies (*Hdp^2^*) contain a point mutation in troponin, a protein required for proper cardiac muscle contraction, and have enlarged diastolic chambers (Wolf et al., 2006). *hdp^2^* mutants display a prolonged duration of the Ca^2+^ peak intensity (see poster), indicating that the late cytosolic Ca^2+^ rise is delayed. This delay, along with protracted Ca^2+^ indicator fluorescence, is speculated to be the result of impaired Ca^2+^ reuptake into the sarcoplasmic reticulum (Lin et al., 2011). Moreover, Ca^2+^ measurements are also useful in the study of age-related cardiac defects, because aging flies display a decrease in the maximal rate of Ca^2+^ fluorescence decay, indicating that spontaneous cardiac frequency is reduced (Santalla et al., 2014). Older flies have variable spontaneous cardiac frequencies, which indicates an arrhythmia in the aging heart. As mentioned previously, elderly humans also demonstrate cardiac arrhythmias. Hence, the result of this study proposes that alterations in Ca^2+^ signaling might be related to arrhythmias observed in the elderly.

### Field potential and intracellular recording

Heart rhythmicity is maintained through electrical conduction. The assays described above to monitor fly heart rhythm do not directly measure electric conduction. For this purpose, electrophysiological methods have been developed to record heart field potential directly, and these approaches can be applied in both larval and adult fly hearts (Papaefthimiou and Theophilidis, 2001). Spontaneous heart field potentials can be measured via a thin glass electrode, which is placed in contact with the semi-intact fly heart (see poster) (Cooper et al., 2009). In larvae, the heart is loosely attached to the body wall, and a floating electrode technique can therefore be used to minimize damage (Lalevée et al., 2006). By contrast, the adult heart adheres more tightly to the body wall, and hence recordings are performed from the myocardium of the heart chamber (Dulcis and Levine, 2005) by stimulating a local glutamatergic input. The action potential recorded from both the larval and adult hearts display a pacemaker potential that is a feature of the myogenic heart. Although these electrophysiological methods are not very convenient for high-throughput screening, they are particularly useful to understand the mechanistic properties of the heart. For example, *KCNQ1* (a gene that encodes a voltage-gated potassium channel) mutant flies display cardiac arrhythmia that worsens with age (Ocorr et al., 2007b). KCNQ1 is partially responsible for the rhythmic contraction of the heart muscle. The electrophysiological recordings from the *KCNQ1* mutant fly hearts show reduced repolarization ability, most probably due to a decrease in the repolarizing K^+^ current (see poster). Interestingly, in humans, mutations in KCNQ1 cause many cardiac disorders, such as familial atrial fibrillation, which is characterized by uncoordinated cardiac electrical activity, and long-QT syndrome, which is also characterized by rapid heartbeats (Bellocq et al., 2004; Johnson et al., 2008). These studies collectively support the use of *Drosophila* as an effective model to study heart disease.

## Oenocyte and fat body assays

Liver disease causes millions of deaths per year worldwide (Byass, 2014). Nonalcoholic fatty liver disease (NAFLD) is the most common form of liver disease, affecting 75-million to 100-million individuals in the USA (Rinella and Sanyal, 2015). Because the burden of this disease is large and costly, the elucidation of pathogenic mechanisms underlying liver disease, using model organisms, is a key healthcare priority. In humans, the liver has many metabolic functions, including detoxification of metabolites, protein synthesis, synthesis of digestive metabolites and maintenance of blood glucose levels. These functions are performed by highly specialized cells named hepatocytes. To regulate fat usage during starvation, adipocytes – the body's major fat-storing cells – break down lipids into fatty acids (FAs) via adipocyte triglyceride lipase (ATGL) (Zechner et al., 2005). The FAs are secreted in the bloodstream, taken up by the liver and processed via hepatocytes. During prolonged starvation, hepatocytes synthesize water-soluble ketone bodies from the FAs, and these are released into the bloodstream to be used as an energy source for other tissues (Green et al., 2015).

Fasting fly larvae similarly release lipids from the fat body – the organ responsible for energy storage and utilization – and these lipids are taken up by specialized cells named oenocytes (Chatterjee et al., 2014). Until recently, the fly fat body was thought to be the functional homolog of the human liver (Baker and Thummel, 2007). However, studies have shown that fly oenocytes are more similar to hepatocytes than is the fat body, based on their response to starvation. Furthermore, oenocytes express 22 homologs of human fat-metabolizing genes expressed in hepatocytes, and also express genes involved in hepatocyte differentiation, including *hepatocyte nuclear factor 4-a* (*Hnf4-a*) and *COUP-transcription factor* (*COUP-TF*) (Gutierrez et al., 2007). Finally, oenocyte-specific knockdown of acetyl-coenzyme A-carboxylase (ACC), a rate-limiting enzyme in FA synthesis, results in lethality, demonstrating the importance of oenocytes for FA synthesis (Parvy et al., 2012). Overall, recent data demonstrate that the fat body and oenocytes in flies are the functional homologs of the vertebrate liver (see poster). In the next section, we will review the fundamental fat body and oenocyte assays to model liver diseases in flies. *Drosophila* has, in our opinion, not been exploited to its full potential as a model system in liver disease research, and there is scope for the development of new assays and the improvement of existing ones.

### Lipid-droplet accumulation

The assays that are used to study fat body and oenocyte function in flies typically depend on visualizing lipid storage in response to differential nutritional conditions. Generally, dyes such as Oil Red-O (Gutierrez et al., 2007) or BODIPY (Kohyama-Koganeya et al., 2008) are used to visualize lipids in oenocytes and the fat body of the fly. An *in vivo* assay to determine the presence of lipids is based on stimulated Raman scattering (SRS) microscopy or coherent anti-Stokes Raman scattering (CARS) microscopy (Chien et al., 2012). These spectroscopic assays detect the vibrational signature of molecules and allows labeling-free, live imaging of lipids (see poster).

Mutations in human HNF4A are associated with a type of inherited diabetes known as maturity-onset diabetes of the young, type 1 (MODY1) (Yamagata et al., 1996). These mutations lead to decreased serum triglyceride (TAG) levels, and the onset of diabetes, in affected individuals (Fajans et al., 2001). The functional homolog of *HNF4A* in flies is *Hnf4. Hnf4*-null flies are very sensitive to starvation because they are unable to harness energy from stored lipids (see poster) (Palanker et al., 2009). Interestingly, many enzymes involved in lipid catabolism are upregulated in starved *Hnf4*-null animals. Investigation of these mutant flies has provided supporting evidence that a specific FA activates the nuclear hormone receptor, which in turn stimulates energy production by activating FA oxidation.

In a forward genetics study, a genome-wide RNAi screen performed in fly oenocytes identified multiple obesity-related genes that are also associated with obesity in mice (Pospisilik et al., 2010). One of the candidates from this screen is the fly homolog of fatty-acid elongase (*ELOVL6*), *baldspot*. Interestingly, mouse mutants of *Elovl6* also develop obesity and hepatosteosis (Matsuzaka et al., 2007). To date, mutations in human *ELOVL6* have not been linked with obesity or liver disease, however. Another obesity model in flies is the fat-body-specific overexpression of *Lipid storage droplet 2* (*Lsd-2*) (Grönke et al., 2003). These flies are obese, resistant to starvation and have elevated TAG storage. Furthermore, CARS microscopy revealed that starved flies that overexpress *Lsd-2* in the fat body have reduced lipids in oenocytes, showing that they are unable to store lipids in oenocytes as a starvation response (see poster) (Chien et al., 2012). The functional homolog of *Lsd-2* in humans is perilipin 2 (*Plin2*) (Rajan and Perrimon, 2013). Perilipins coat intracellular lipid droplets and are involved in lipolysis. Interestingly, the liver biopsies from nonalcoholic steatohepatitis patients show increased levels of PLIN2 in lipid droplets, indicating the importance of *Plin2* for individuals with NAFLD (Fujii et al., 2009). In addition to genetic risk factors, environmental factors that result in liver disease can also be studied in flies. Obesity and type 2 diabetes are the major risk factors for NAFLD (Neuschwander-Tetri et al., 2010). Fly larvae that are fed with a high-sugar diet mimic the hallmarks of both obesity and type 2 diabetes (Owusu-Ansah and Perrimon, 2014). The high-sugar-fed larvae have increased body fat along with the accumulation of large lipid droplets in the fat bodies (Musselman et al., 2011). The transcriptional profile of these larvae demonstrates an increase in the expression of genes related to lipid catabolism. Of note, the transcriptional profile of these larvae is similar to *Hnf4*-null flies, indicating a conserved transcriptional signature that might be instrumental in diabetes and liver disease. In summary, these results provide compelling evidence that the fly can help provide a valuable mechanistic understanding of processes that cause liver diseases.

## Nephrocyte and Malpighian tubule assays

The function of the human excretory system is to eliminate metabolic waste and maintain a homeostatic ion balance. The nephron is the basic structural and functional unit of the human kidney. It is composed of a glomerulus, glomerular capsule and renal tube. The glomerular podocyte is an epithelial cell that wraps around capillaries in the glomerulus and plays an important role in the filtration of blood to produce urine. Glomerular podocytes create the filtration barrier by sending out interdigitating processes that are separated by 30- to 50-nm-wide slit pores, called the slit diaphragm (see poster) (Wartiovaara et al., 2004). Blood is filtered through these slit diaphragms, and mutations in several genes that disrupt the filtration barrier lead to kidney failure (Kestilä et al., 1998; Patrakka et al., 2000). Although invertebrates lack nephrons, *Drosophila* nephrocytes share remarkable similarity to podocytes. There are two different nephrocytes in *Drosophila*: the pericardial nephrocytes, which flank the fly aorta, and garland nephrocytes, which form a ring around the proventriculus (Denholm et al., 2013). These nephrocytes exhibit extensive folds of the plasma membrane and create ∼30-nm slit pores (Weavers et al., 2009), forming the nephrocyte diaphragm, which shares functional and molecular similarities with the slit diaphragm of the mammalian podocyte (see poster). Indeed, similar to the mammalian podocyte, the proteins encoded by *sticks and stones* (*sns*) and *dumbfounded* (*duf*), the *Drosophila* homologs of nephrosis 1 (*NPHS1*) and nephrin 1 (*NEPH1*), form homo- and heterotypic interactions across the slit pore of nephrocytes in flies (Weavers et al., 2009).

Besides nephrocytes, the fly uses Malpighian tubules to clear toxins, produce uric acid, regulate ions and acid-bases, and balance fluid (Beyenbach et al., 2010). Again, many genes, such as the vacuolar-type-ATPases (V-ATPases), Na^+^/K^+^-ATPase, aquaporins and several ion channels and transporters, are shared between flies and mammals and are involved in ion homeostasis (Wang et al., 2004). Interestingly, mutations in the *rosy* (*ry*) and *maroon-like* genes in fly lead to a sensitivity to dietary purines and bloated, malformed Malpighian tubules (Hadorn and Schwinck, 1956). *ry* encodes a xanthine oxidase, and its deficiency in humans causes type I xanthinuria (an inborn error of metabolism) (Dent and Philpot, 1954). Biochemical studies of the fly mutants have provided a clear understanding of the source of the disease: accumulation of xanthine, the *ry* enzyme's substrate (Bonse, 1967; Glassman and Mitchell, 1959; Kamleh et al., 2008; Mitchell and Glassman, 1959). In addition, recently, Malpighian tubules have been used to model kidney-stone formation. Because the tubules are transparent, they enable observation of stone nucleation and growth of oxalate crystals in flies (Hirata et al., 2012; Landry et al., 2015).

### Nephrocyte filtration assay

The nephrocyte filtration assay is based on the nephrocyte's ability to take up fluorescently labeled dextrans with different molecular masses: nephrocytes filter the hemolymph with a size-dependent efficiency (see poster) (Weavers et al., 2009). By using this assay as well as other approaches, two key genes were identified: *sns* and *duf*. Mutations in human homologs of *sns* cause congenital nephrotic syndrome (Kestilä et al., 1998), whereas loss of the *duf* homolog in mice disrupts the slit diaphragm and causes nephrotic syndrome at birth (Donoviel et al., 2001; Liu et al., 2003).

The nephrocyte filtration assay is a convenient assay to identify new genes in forward genetic screens. Indeed, an RNAi screen based on a modified filtration assay identified many genes that are required for nephrocyte function (Zhang et al., 2013a). Instead of dextrans, the authors overexpressed a secreted fluorescent peptide, rat atrial natriuretic factor-GFP (ANF-GFP) from muscle and observed the uptake of the peptide in pericardial nephrocytes (see poster). Several genes that are required for nephrocyte filtration function were identified via this preliminary screen. The human homologs of these genes are linked to renal diseases, including *mec2*, *CG11592* (the *Drosophila* homolog of mammalian amnionless) and *CG32702* (the *Drosophila* homolog of mammalian cubilin) (Boute et al., 2000; Storm et al., 2011; Wahlstedt-Froberg et al., 2003).

The function of Malpighian tubules can be determined by visually assessing their transparency (not shown in poster). For example, loss-of-function mutations in the V-ATPase (*vha55*), a transmembrane protein required for proton transport, was identified via this assay in *Drosophila* (Davies et al., 1996). It was then observed that every mutation in a gene that encodes subunits of the V-ATPase complex leads to a transparent Malpighian tubule, indicating a defect in acidification (Allan et al., 2005; Davies et al., 1996; Dow, 1999). Later, a mutation in the human B_1_ subunit of the V-ATPase was discovered to cause renal tubular acidosis (Karet et al., 1999). In summary, the *Drosophila* nephrocytes and Malpighian tubules have been shown to be valuable models in the study of human renal diseases.

## Concluding remarks

*Drosophila* provide a powerful platform to perform functional annotations of human genes and disease variants, given the observation that evolutionarily conserved genes tend to have similar molecular functions. The fly community is continually providing state-of-the-art tools and resources that are rapidly evolving and permit efficient gene and genome engineering. The assays described here permit evaluation of how these genes affect specific cellular processes and allow us to study the molecular mechanisms that underlie diseases of the nervous, cardiovascular, metabolic and renal systems, and beyond. Furthermore, the assays that can be used in genetic screens should allow us to uncover as-yet-uncharacterized disease-causing genes.

## References

[DMM023762C1] Aberle H., Haghighi A. P., Fetter R. D., McCabe B. D., Magalhães T. R. and Goodman C. S. (2002). wishful thinking encodes a BMP type II receptor that regulates synaptic growth in Drosophila. *Neuron* 33, 545-558. 10.1016/S0896-6273(02)00589-5 11856529

[DMM023762C2] Allan A. K., Du J., Davies S. A. and Dow J. A. T. (2005). Genome-wide survey of V-ATPase genes in Drosophila reveals a conserved renal phenotype for lethal alleles. *Physiol. Genomics* 22, 128-138. 10.1152/physiolgenomics.00233.2004 15855386

[DMM023762C3] Andlauer T. F. M. and Sigrist S. J. (2012). In vivo imaging of Drosophila larval neuromuscular junctions to study synapse assembly. *Cold Spring Harb. Protoc.* 2012, 407-413. 10.1101/pdb.top068577 22474662

[DMM023762C4] Apidianakis Y. and Rahme L. G. (2011). Drosophila melanogaster as a model for human intestinal infection and pathology. *Dis. Model. Mech.* 4, 21-30. 10.1242/dmm.003970 21183483 PMC3014343

[DMM023762C5] Baker K. D. and Thummel C. S. (2007). Diabetic larvae and obese flies-emerging studies of metabolism in Drosophila. *Cell Metab.* 6, 257-266. 10.1016/j.cmet.2007.09.002 17908555 PMC2231808

[DMM023762C6] Bayat V., Thiffault I., Jaiswal M., Tétreault M., Donti T., Sasarman F., Bernard G., Demers-Lamarche J., Dicaire M.-J., Mathieu J. et al. (2012). Mutations in the mitochondrial methionyl-tRNA synthetase cause a neurodegenerative phenotype in flies and a recessive ataxia (ARSAL) in humans. *PLoS Biol.* 10, e1001288 10.1371/journal.pbio.1001288 22448145 PMC3308940

[DMM023762C7] Bellen H. J. and Yamamoto S. (2015). Morgan's legacy: fruit flies and the functional annotation of conserved genes. *Cell* 163, 12-14. 10.1016/j.cell.2015.09.009 26406362 PMC4783153

[DMM023762C8] Bellocq C., van Ginneken A. C. G., Bezzina C. R., Alders M., Escande D., Mannens M. M. A. M., Baro I. and Wilde A. A. M. (2004). Mutation in the KCNQ1 gene leading to the short QT-interval syndrome. *Circulation* 109, 2394-2397. 10.1161/01.CIR.0000130409.72142.FE 15159330

[DMM023762C9] Beumer K. J. and Carroll D. (2014). Targeted genome engineering techniques in Drosophila. *Methods* 68, 29-37. 10.1016/j.ymeth.2013.12.002 24412316 PMC4048800

[DMM023762C10] Beyenbach K. W., Skaer H. and Dow J. A. T. (2010). The developmental, molecular, and transport biology of Malpighian tubules. *Annu. Rev. Entomol.* 55, 351-374. 10.1146/annurev-ento-112408-085512 19961332

[DMM023762C11] Bier E. and Bodmer R. (2004). Drosophila, an emerging model for cardiac disease. *Gene* 342, 1-11. 10.1016/j.gene.2004.07.018 15527959

[DMM023762C12] Bodmer R. (1993). The gene tinman is required for specification of the heart and visceral muscles in Drosophila. *Development* 118, 719-729. 7915669 10.1242/dev.118.3.719

[DMM023762C13] Bonse A. (1967). [Studies on the chemical nature and formation of the urinary conglomerate in the Malpighian vessels of the rosy mutant of Drosophila melanogaster]. *Z. Naturforsch.* 22, 1027-1029. 10.1515/znb-1967-1008 4385816

[DMM023762C14] Boute N., Gribouval O., Roselli S., Benessy F., Lee H., Fuchshuber A., Dahan K., Gubler M.-C., Niaudet P. and Antignac C. (2000). NPHS2, encoding the glomerular protein podocin, is mutated in autosomal recessive steroid-resistant nephrotic syndrome. *Nat. Genet.* 24, 349-354. 10.1038/74166 10742096

[DMM023762C15] Branson K., Robie A. A., Bender J., Perona P. and Dickinson M. H. (2009). High-throughput ethomics in large groups of Drosophila. *Nat. Methods* 6, 451-457. 10.1038/nmeth.1328 19412169 PMC2734963

[DMM023762C16] Broadie K. S. and Bate M. (1993). Development of the embryonic neuromuscular synapse of Drosophila melanogaster. *J. Neurosci.* 13, 144-166. 8093713 10.1523/JNEUROSCI.13-01-00144.1993 PMC6576301

[DMM023762C17] Byass P. (2014). The global burden of liver disease: a challenge for methods and for public health. *BMC Med.* 12, 159 10.1186/s12916-014-0159-5 25286285 PMC4168048

[DMM023762C18] Chakraborty M., Selma-Soriano E., Magny E., Couso J. P., Pérez-Alonso M., Charlet-Berguerand N., Artero R. and Llamusi B. (2015). Pentamidine rescues contractility and rhythmicity in a Drosophila model of myotonic dystrophy heart dysfunction. *Dis. Model. Mech.* 8, 1569-1578. 10.1242/dmm.021428 26515653 PMC4728315

[DMM023762C19] Charng W.-L., Yamamoto S. and Bellen H. J. (2014). Shared mechanisms between Drosophila peripheral nervous system development and human neurodegenerative diseases. *Curr. Opin. Neurobiol.* 27, 158-164. 10.1016/j.conb.2014.03.001 24762652 PMC4122633

[DMM023762C20] Chatterjee D., Katewa S. D., Qi Y., Jackson S. A., Kapahi P. and Jasper H. (2014). Control of metabolic adaptation to fasting by dILP6-induced insulin signaling in Drosophila oenocytes. *Proc. Natl. Acad. Sci. USA* 111, 17959-17964. 10.1073/pnas.1409241111 25472843 PMC4273364

[DMM023762C21] Chien S., Reiter L. T., Bier E. and Gribskov M. (2002). Homophila: human disease gene cognates in Drosophila. *Nucleic Acids Res.* 30, 149-151. 10.1093/nar/30.1.149 11752278 PMC99119

[DMM023762C22] Chien C.-H., Chen W.-W., Wu J.-T. and Chang T.-C. (2012). Investigation of lipid homeostasis in living Drosophila by coherent anti-Stokes Raman scattering microscopy. *J. Biomed. Opt.* 17, 126001 10.1117/1.JBO.17.12.126001 23208212

[DMM023762C23] Chintapalli V. R., Wang J. and Dow J. A. T. (2007). Using FlyAtlas to identify better Drosophila melanogaster models of human disease. *Nat. Genet.* 39, 715-720. 10.1038/ng2049 17534367

[DMM023762C24] Choma M. A., Suter M. J., Vakoc B. J., Bouma B. E. and Tearney G. J. (2011). Physiological homology between Drosophila melanogaster and vertebrate cardiovascular systems. *Dis. Model. Mech.* 4, 411-420. 10.1242/dmm.005231 21183476 PMC3097462

[DMM023762C25] Cook R. K., Christensen S. J., Deal J. A., Coburn R. A., Deal M. E., Gresens J. M., Kaufman T. C. and Cook K. R. (2012). The generation of chromosomal deletions to provide extensive coverage and subdivision of the Drosophila melanogaster genome. *Genome Biol.* 13, R21 10.1186/gb-2012-13-3-r21 22445104 PMC3439972

[DMM023762C26] Cooper A. S., Rymond K. E., Ward M. A., Bocook E. L. and Cooper R. L. (2009). Monitoring heart function in larval Drosophila melanogaster for physiological studies. *J. Vis. Exp.* e1596 10.3791/1596 PMC3353715 19918216

[DMM023762C27] Davies S. A., Goodwin S. F., Kelly D. C., Wang Z., Sozen M. A., Kaiser K. and Dow J. A. T. (1996). Analysis and inactivation of vha55, the gene encoding the vacuolar ATPase B-subunit in Drosophila melanogaster reveals a larval lethal phenotype. *J. Biol. Chem.* 271, 30677-30684. 10.1074/jbc.271.48.30677 8940044

[DMM023762C28] de la Pompa J. L. and Epstein J. A. (2012). Coordinating tissue interactions: Notch signaling in cardiac development and disease. *Dev. Cell* 22, 244-254. 10.1016/j.devcel.2012.01.014 22340493 PMC3285259

[DMM023762C29] Denholm B., Hu N., Fauquier T., Caubit X., Fasano L. and Skaer H. (2013). The tiptop/teashirt genes regulate cell differentiation and renal physiology in Drosophila. *Development* 140, 1100-1110. 10.1242/dev.088989 23404107 PMC3583044

[DMM023762C30] Dent C. E. and Philpot G. R. (1954). Xanthinuria, an inborn error (or deviation) of metabolism. *Lancet* 263, 182-185. 10.1016/S0140-6736(54)91257-X 13118765

[DMM023762C31] Diop S. B. and Bodmer R. (2015). Gaining insights into diabetic cardiomyopathy from Drosophila. *Trends Endocrinol. Metab.* 26, 618-627. 10.1016/j.tem.2015.09.009 26482877 PMC4638170

[DMM023762C32] Donoviel D. B., Freed D. D., Vogel H., Potter D. G., Hawkins E., Barrish J. P., Mathur B. N., Turner C. A., Geske R., Montgomery C. A. et al. (2001). Proteinuria and perinatal lethality in mice lacking NEPH1, a novel protein with homology to NEPHRIN. *Mol. Cell. Biol.* 21, 4829-4836. 10.1128/MCB.21.14.4829-4836.2001 11416156 PMC87176

[DMM023762C33] Dow J. A. T. (1999). The multifunctional Drosophila melanogaster V-ATPase is encoded by a multigene family. *J. Bioenerg. Biomembr.* 31, 75-84. 10.1023/A:1005400731289 10340851

[DMM023762C34] Dulcis D. and Levine R. B. (2005). Glutamatergic innervation of the heart initiates retrograde contractions in adult Drosophila melanogaster. *J. Neurosci.* 25, 271-280. 10.1523/JNEUROSCI.2906-04.2005 15647470 PMC6725498

[DMM023762C35] Dutta S., Rieche F., Eckl N., Duch C. and Kretzschmar D. (2016). Glial expression of Swiss cheese (SWS), the Drosophila orthologue of neuropathy target esterase (NTE), is required for neuronal ensheathment and function. *Dis. Model. Mech.* 9, 283-294. 10.1242/dmm.022236 26634819 PMC4826977

[DMM023762C36] Escayg A., MacDonald B. T., Meisler M. H., Baulac S., Huberfeld G., An-Gourfinkel I., Brice A., LeGuern E., Moulard B., Chaigne D. et al. (2000). Mutations of SCN1A, encoding a neuronal sodium channel, in two families with GEFS+2. *Nat. Genet.* 24, 343-345. 10.1038/74159 10742094

[DMM023762C37] Fajans S. S., Bell G. I. and Polonsky K. S. (2001). Molecular mechanisms and clinical pathophysiology of maturity-onset diabetes of the young. *N. Engl. J. Med.* 345, 971-980. 10.1056/NEJMra002168 11575290

[DMM023762C38] Fang Y., Soares L., Teng X., Geary M. and Bonini N. M. (2012). A novel Drosophila model of nerve injury reveals an essential role of Nmnat in maintaining axonal integrity. *Curr. Biol.* 22, 590-595. 10.1016/j.cub.2012.01.065 22425156 PMC3347919

[DMM023762C39] Feany M. B. and Bender W. W. (2000). A Drosophila model of Parkinson's disease. *Nature* 404, 394-398. 10.1038/35006074 10746727

[DMM023762C40] Feng G., Deak P., Chopra M. and Hall L. M. (1995). Cloning and functional analysis of TipE, a novel membrane protein that enhances Drosophila para sodium channel function. *Cell* 82, 1001-1011. 10.1016/0092-8674(95)90279-1 7553842

[DMM023762C41] Ferrari R. and Rizzo P. (2014). The Notch pathway: a novel target for myocardial remodelling therapy? *Eur. Heart J.* 35, 2140-2145. 10.1093/eurheartj/ehu244 24970336

[DMM023762C42] Fink M., Callol-Massot C., Chu A., Ruiz-Lozano P., Izpisua Belmonte J. C., Giles W., Bodmer R. and Ocorr K. (2009). A new method for detection and quantification of heartbeat parameters in Drosophila, zebrafish, and embryonic mouse hearts. *BioTechniques* 46, 101-113. 10.2144/000113078 19317655 PMC2855226

[DMM023762C43] Fujii H., Ikura Y., Arimoto J., Sugioka K., Iezzoni J. C., Park S. H., Naruko T., Itabe H., Kawada N., Caldwell S. H. et al. (2009). Expression of perilipin and adipophilin in nonalcoholic fatty liver disease; relevance to oxidative injury and hepatocyte ballooning. *J. Atheroscler. Thromb.* 16, 893-901. 10.5551/jat.2055 20032580

[DMM023762C44] Garg V., Muth A. N., Ransom J. F., Schluterman M. K., Barnes R., King I. N., Grossfeld P. D. and Srivastava D. (2005). Mutations in NOTCH1 cause aortic valve disease. *Nature* 437, 270-274. 10.1038/nature03940 16025100

[DMM023762C45] Gill S., Le H. D., Melkani G. C. and Panda S. (2015). Time-restricted feeding attenuates age-related cardiac decline in Drosophila. *Science* 347, 1265-1269. 10.1126/science.1256682 25766238 PMC4578815

[DMM023762C46] Glassman E. and Mitchell H. K. (1959). Mutants of Drosophila melanogaster deficient in xanthine dehydrogenase. *Genetics* 44, 153-162. 17247815 10.1093/genetics/44.2.153 PMC1209939

[DMM023762C47] Green C. J., Pramfalk C., Morten K. J. and Hodson L. (2015). From whole body to cellular models of hepatic triglyceride metabolism: man has got to know his limitations. *Am. J. Physiol. Endocrinol. Metab.* 308, E1-E20. 10.1152/ajpendo.00192.2014 25352434 PMC4281685

[DMM023762C48] Grönke S., Beller M., Fellert S., Ramakrishnan H., Jäckle H. and Kühnlein R. P. (2003). Control of fat storage by a Drosophila PAT domain protein. *Curr. Biol.* 13, 603-606. 10.1016/S0960-9822(03)00175-1 12676093

[DMM023762C49] Gu G.-G. and Singh S. (1995). Pharmacological analysis of heartbeat in Drosophila. *J. Neurobiol.* 28, 269-280. 10.1002/neu.480280302 8568510

[DMM023762C50] Guo T., Zhang T., Mestril R. and Bers D. M. (2006). Ca2+/Calmodulin-dependent protein kinase II phosphorylation of ryanodine receptor does affect calcium sparks in mouse ventricular myocytes. *Circ. Res.* 99, 398-406. 10.1161/01.RES.0000236756.06252.13 16840718

[DMM023762C51] Gutierrez E., Wiggins D., Fielding B. and Gould A. P. (2007). Specialized hepatocyte-like cells regulate Drosophila lipid metabolism. *Nature* 445, 275-280. 10.1038/nature05382 17136098

[DMM023762C52] Hadorn E. and Schwinck I. (1956). [A mutant (rosy2) of Drosophila melanogaster without isoxanthopterin which is non-autonomous for the red eye pigments]. *Z. Induktive Abstamm. Vererbungsl.* 87, 528-553. 13393172

[DMM023762C53] Haelterman N. A., Jiang L., Li Y., Bayat V., Sandoval H., Ugur B., Tan K. L., Zhang K., Bei D., Xiong B. et al. (2014). Large-scale identification of chemically induced mutations in Drosophila melanogaster. *Genome Res.* 24, 1707-1718. 10.1101/gr.174615.114 25258387 PMC4199363

[DMM023762C54] Hardie R. C. and Raghu P. (2001). Visual transduction in Drosophila. *Nature* 413, 186-193. 10.1038/35093002 11557987

[DMM023762C55] Herrmann D. N., Horvath R., Sowden J. E., Gonzales M., Sanchez-Mejias A., Guan Z., Whittaker R. G., Almodovar J. L., Lane M., Bansagi B. et al. (2014). Synaptotagmin 2 mutations cause an autosomal-dominant form of lambert-eaton myasthenic syndrome and nonprogressive motor neuropathy. *Am. J. Hum. Genet.* 95, 332-339. 10.1016/j.ajhg.2014.08.007 25192047 PMC4157148

[DMM023762C56] Hirata T., Czapar A., Brin L., Haritonova A., Bondeson D. P., Linser P., Cabrero P., Thompson J., Dow J. A. T. and Romero M. F. (2012). Ion and solute transport by Prestin in Drosophila and Anopheles. *J. Insect Physiol.* 58, 563-569. 10.1016/j.jinsphys.2012.01.009 22321763 PMC3482613

[DMM023762C57] Hotta Y. and Benzer S. (1969). Abnormal electroretinograms in visual mutants of Drosophila. *Nature* 222, 354-356. 10.1038/222354a0 5782111

[DMM023762C58] Inagaki H. K., Kamikouchi A. and Ito K. (2010). Protocol for quantifying sound-sensing ability of Drosophila melanogaster. *Nat. Protoc.* 5, 26-30. 10.1038/nprot.2009.206 20010725

[DMM023762C59] Jan L. Y. and Jan Y. N. (1976). Properties of the larval neuromuscular junction in Drosophila melanogaster. *J. Physiol.* 262, 189-214. 10.1113/jphysiol.1976.sp011592 11339 PMC1307637

[DMM023762C60] Jan Y. N., Jan L. Y. and Dennis M. J. (1977). Two mutations of synaptic transmission in Drosophila. *Proc. R. Soc. Lond. B Biol. Sci.* 198, 87-108. 10.1098/rspb.1977.0087 20636

[DMM023762C61] Jentsch T. J. (2000). Neuronal KCNQ potassium channels: physiology and role in disease. *Nat. Rev.* 1, 21-30. 10.1038/35036198 11252765

[DMM023762C62] Johnson J. N., Tester D. J., Perry J., Salisbury B. A., Reed C. R. and Ackerman M. J. (2008). Prevalence of early-onset atrial fibrillation in congenital long QT syndrome. *Heart Rhythm* 5, 704-709. 10.1016/j.hrthm.2008.02.007 18452873 PMC3940082

[DMM023762C63] Jones S. A. (2006). Ageing to arrhythmias: conundrums of connections in the ageing heart. *J. Pharm. Pharmacol.* 58, 1571-1576. 10.1211/jpp.58.12.0002 17331319

[DMM023762C64] Kamleh M. A., Hobani Y., Dow J. A. T. and Watson D. G. (2008). Metabolomic profiling of Drosophila using liquid chromatography Fourier transform mass spectrometry. *FEBS Lett.* 582, 2916-2922. 10.1016/j.febslet.2008.07.029 18657538

[DMM023762C65] Kaplan W. D. and Trout W. E. III (1969). The behavior of four neurological mutants of Drosophila. *Genetics* 61, 399-409. 5807804 10.1093/genetics/61.2.399 PMC1212165

[DMM023762C66] Karet F. E., Finberg K. E., Nelson R. D., Nayir A., Mocan H., Sanjad S. A., Rodriguez-Soriano J., Santos F., Cremers C. W. R. J., Di Pietro A. et al. (1999). Mutations in the gene encoding B1 subunit of H+-ATPase cause renal tubular acidosis with sensorineural deafness. *Nat. Genet.* 21, 84-90. 10.1038/5022 9916796

[DMM023762C67] Kerr F., Augustin H., Piper M. D. W., Gandy C., Allen M. J., Lovestone S. and Partridge L. (2011). Dietary restriction delays aging, but not neuronal dysfunction, in Drosophila models of Alzheimer's disease. *Neurobiol. Aging* 32, 1977-1989. 10.1016/j.neurobiolaging.2009.10.015 19969390 PMC3176895

[DMM023762C68] Kestilä M., Lenkkeri U., Männikkö M., Lamerdin J., McCready P., Putaala H., Ruotsalainen V., Morita T., Nissinen M., Herva R. et al. (1998). Positionally cloned gene for a novel glomerular protein—nephrin—is mutated in congenital nephrotic syndrome. *Mol. Cell* 1, 575-582. 10.1016/S1097-2765(00)80057-X 9660941

[DMM023762C69] Kim I. M., Wolf M. J. and Rockman H. A. (2010). Gene deletion screen for cardiomyopathy in adult Drosophila identifies a new notch ligand. *Circ. Res.* 106, 1233-1243. 10.1161/CIRCRESAHA.109.213785 20203305 PMC2860286

[DMM023762C70] King D. G. and Wyman R. J. (1980). Anatomy of the giant fibre pathway in Drosophila. I. Three thoracic components of the pathway. *J. Neurocytol.* 9, 753-770. 10.1007/BF01205017 6782199

[DMM023762C71] Kohyama-Koganeya A., Kim Y.-J., Miura M. and Hirabayashi Y. (2008). A Drosophila orphan G protein-coupled receptor BOSS functions as a glucose-responding receptor: loss of boss causes abnormal energy metabolism. *Proc. Natl. Acad. Sci. USA* 105, 15328-15333. 10.1073/pnas.0807833105 18832180 PMC2563099

[DMM023762C72] Kurdyak P., Atwood H. L., Stewart B. A. and Wu C.-F. (1994). Differential physiology and morphology of motor axons to ventral longitudinal muscles in larval Drosophila. *J. Comp. Neurol.* 350, 463-472. 10.1002/cne.903500310 7884051

[DMM023762C73] Lalevée N., Monier B., Sénatore S., Perrin L. and Sémériva M. (2006). Control of cardiac rhythm by ORK1, a Drosophila two-pore domain potassium channel. *Curr. Biol.* 16, 1502-1508. 10.1016/j.cub.2006.05.064 16890525

[DMM023762C74] Landry G. M., Hirata T., Anderson J. B., Cabrero P., Gallo C. J. R., Dow J. A. T. and Romero M. F. (2015). Sulfate and thiosulfate inhibit oxalate transport via a dPrestin(mSlc26a6)-dependent mechanism in an insect model of calcium oxalate nephrolithiasis. *Am. J. Physiol.* 310, F152-F159. 10.1152/ajprenal.00406.2015 PMC4719044 26538444

[DMM023762C75] Lee W.-C. M., Yoshihara M. and Littleton J. T. (2004). Cytoplasmic aggregates trap polyglutamine-containing proteins and block axonal transport in a Drosophila model of Huntington's disease. *Proc. Natl. Acad. Sci. USA* 101, 3224-3229. 10.1073/pnas.0400243101 14978262 PMC365771

[DMM023762C76] Lehmacher C., Abeln B. and Paululat A. (2012). The ultrastructure of Drosophila heart cells. *Arthropod. Struct. Dev.* 41, 459-474. 10.1016/j.asd.2012.02.002 22426062

[DMM023762C77] Lin N., Badie N., Yu L., Abraham D., Cheng H., Bursac N., Rockman H. A. and Wolf M. J. (2011). A method to measure myocardial calcium handling in adult Drosophila. *Circ. Res.* 108, 1306-1315. 10.1161/CIRCRESAHA.110.238105 21493892 PMC3128985

[DMM023762C78] Littleton J. T., Stern M., Perin M. and Bellen H. J. (1994). Calcium dependence of neurotransmitter release and rate of spontaneous vesicle fusions are altered in Drosophila synaptotagmin mutants. *Proc. Natl. Acad. Sci. USA* 91, 10888-10892. 10.1073/pnas.91.23.10888 7971978 PMC45131

[DMM023762C79] Liu G., Kaw B., Kurfis J., Rahmanuddin S., Kanwar Y. S. and Chugh S. S. (2003). Neph1 and nephrin interaction in the slit diaphragm is an important determinant of glomerular permeability. *J. Clin. Invest.* 112, 209-221. 10.1172/JCI200318242 12865409 PMC164293

[DMM023762C80] Liu L., Zhang K., Sandoval H., Yamamoto S., Jaiswal M., Sanz E., Li Z., Hui J., Graham B. H., Quintana A. et al. (2015). Glial lipid droplets and ROS induced by mitochondrial defects promote neurodegeneration. *Cell* 160, 177-190. 10.1016/j.cell.2014.12.019 25594180 PMC4377295

[DMM023762C81] Macleod G. T. (2012). Calcium imaging at the Drosophila larval neuromuscular junction. *Cold Spring Harb. Protoc.* 2012, 758-766. 10.1101/pdb.top070078 22753609

[DMM023762C82] Matsuzaka T., Shimano H., Yahagi N., Kato T., Atsumi A., Yamamoto T., Inoue N., Ishikawa M., Okada S., Ishigaki N. et al. (2007). Crucial role of a long-chain fatty acid elongase, Elovl6, in obesity-induced insulin resistance. *Nat. Med.* 13, 1193-1202. 10.1038/nm1662 17906635

[DMM023762C83] McGuire S. E., Deshazer M. and Davis R. L. (2005). Thirty years of olfactory learning and memory research in Drosophila melanogaster. *Prog. Neurobiol.* 76, 328-347. 10.1016/j.pneurobio.2005.09.003 16266778

[DMM023762C84] Meisler M. H. and Kearney J. A. (2005). Sodium channel mutations in epilepsy and other neurological disorders. *J. Clin. Invest.* 115, 2010-2017. 10.1172/JCI25466 16075041 PMC1180547

[DMM023762C85] Mitchell H. K. and Glassman E. (1959). Hypoxanthine in rosy and maroon-like mutants of Drosophila melanogaster. *Science* 129, 268 10.1126/science.129.3344.268 13624714

[DMM023762C86] Mohr S. E. (2014). RNAi screening in Drosophila cells and in vivo. *Methods* 68, 82-88. 10.1016/j.ymeth.2014.02.018 24576618 PMC4206080

[DMM023762C87] Musselman L. P., Fink J. L., Narzinski K., Ramachandran P. V., Hathiramani S. S., Cagan R. L. and Baranski T. J. (2011). A high-sugar diet produces obesity and insulin resistance in wild-type Drosophila. *Dis. Model. Mech.* 4, 842-849. 10.1242/dmm.007948 21719444 PMC3209653

[DMM023762C88] Nahm M., Kim S., Paik S. K., Lee M., Lee S., Lee Z. H., Kim J., Lee D., Bae Y. C. and Lee S. (2010). dCIP4 (Drosophila Cdc42-interacting protein 4) restrains synaptic growth by inhibiting the secretion of the retrograde Glass bottom boat signal. *J. Neurosci.* 30, 8138-8150. 10.1523/JNEUROSCI.0256-10.2010 20554864 PMC6634589

[DMM023762C89] Neukomm L. J., Burdett T. C., Gonzalez M. A., Züchner S. and Freeman M. R. (2014). Rapid in vivo forward genetic approach for identifying axon death genes in Drosophila. *Proc. Natl. Acad. Sci. USA* 111, 9965-9970. 10.1073/pnas.1406230111 24958874 PMC4103363

[DMM023762C90] Neuschwander-Tetri B. A., Clark J. M., Bass N. M., Van Natta M. L., Unalp-Arida A., Tonascia J., Zein C. O., Brunt E. M., Kleiner D. E., McCullough A. J. et al. (2010). Clinical, laboratory and histological associations in adults with nonalcoholic fatty liver disease. *Hepatology* 52, 913-924. 10.1002/hep.23784 20648476 PMC3070295

[DMM023762C91] Newsome T. P., Asling B. and Dickson B. J. (2000). Analysis of Drosophila photoreceptor axon guidance in eye-specific mosaics. *Development* 127, 851-860. 10648243 10.1242/dev.127.4.851

[DMM023762C92] Ocorr K., Akasaka T. and Bodmer R. (2007a). Age-related cardiac disease model of Drosophila. *Mech. Ageing Dev.* 128, 112-116. 10.1016/j.mad.2006.11.023 17125816 PMC1850850

[DMM023762C93] Ocorr K., Reeves N. L., Wessells R. J., Fink M., Chen H.-S. V., Akasaka T., Yasuda S., Metzger J. M., Giles W., Posakony J. W. et al. (2007b). KCNQ potassium channel mutations cause cardiac arrhythmias in Drosophila that mimic the effects of aging. *Proc. Natl. Acad. Sci. USA* 104, 3943-3948. 10.1073/pnas.0609278104 17360457 PMC1820688

[DMM023762C94] Ocorr K., Fink M., Cammarato A., Bernstein S. I. and Bodmer R. (2009). Semi-automated Optical Heartbeat Analysis of small hearts. *J. Vis. Exp.* e1435 10.3791/1435 PMC3150057 19759521

[DMM023762C95] Ocorr K., Vogler G. and Bodmer R. (2014). Methods to assess Drosophila heart development, function and aging. *Methods* 68, 265-272. 10.1016/j.ymeth.2014.03.031 24727147 PMC4058868

[DMM023762C96] Owusu-Ansah E. and Perrimon N. (2014). Modeling metabolic homeostasis and nutrient sensing in Drosophila: implications for aging and metabolic diseases. *Dis. Model. Mech.* 7, 343-350. 10.1242/dmm.012989 24609035 PMC3944494

[DMM023762C97] Pak W. L., Grossfield J. and White N. V. (1969). Nonphototactic mutants in a study of vision of Drosophila. *Nature* 222, 351-354. 10.1038/222351a0 5782110

[DMM023762C98] Palanker L., Tennessen J. M., Lam G. and Thummel C. S. (2009). Drosophila HNF4 regulates lipid mobilization and beta-oxidation. *Cell Metab.* 9, 228-239. 10.1016/j.cmet.2009.01.009 19254568 PMC2673486

[DMM023762C99] Papaefthimiou C. and Theophilidis G. (2001). An in vitro method for recording the electrical activity of the isolated heart of the adult Drosophila melanogaster. *In Vitro Cell Dev. Biol. Anim.* 37, 445-449. 10.1290/1071-2690(2001)037<0445:AIVMFR>2.0.CO;2 11573820

[DMM023762C100] Parker L., Padilla M., Du Y., Dong K. and Tanouye M. A. (2011). Drosophila as a model for epilepsy: bss is a gain-of-function mutation in the para sodium channel gene that leads to seizures. *Genetics* 187, 523-534. 10.1534/genetics.110.123299 21115970 PMC3030494

[DMM023762C101] Parvy J.-P., Napal L., Rubin T., Poidevin M., Perrin L., Wicker-Thomas C. and Montagne J. (2012). Drosophila melanogaster Acetyl-CoA-carboxylase sustains a fatty acid-dependent remote signal to waterproof the respiratory system. *PLoS Genet.* 8, e1002925 10.1371/journal.pgen.1002925 22956916 PMC3431307

[DMM023762C102] Patrakka J., Kestilä M., Wartiovaara J., Ruotsalainen V., Tissari P., Lenkkeri U., Männikkö M., Visapää I., Holmberg C., Rapola J. et al. (2000). Congenital nephrotic syndrome (NPHS1): features resulting from different mutations in Finnish patients. *Kidney Int.* 58, 972-980. 10.1046/j.1523-1755.2000.00254.x 10972661

[DMM023762C103] Pavlidis P. and Tanouye M. A. (1995). Seizures and failures in the giant fiber pathway of Drosophila bang-sensitive paralytic mutants. *J. Neurosci.* 15, 5810-5819. 7643221 10.1523/JNEUROSCI.15-08-05810.1995 PMC6577637

[DMM023762C104] Pavlidis P., Ramaswami M. and Tanouye M. A. (1994). The Drosophila easily shocked gene: a mutation in a phospholipid synthetic pathway causes seizure, neuronal failure, and paralysis. *Cell* 79, 23-33. 10.1016/0092-8674(94)90397-2 7923374

[DMM023762C105] Pennetta G., Hiesinger P. R., Fabian-Fine R., Meinertzhagen I. A. and Bellen H. J. (2002). Drosophila VAP-33A directs bouton formation at neuromuscular junctions in a dosage-dependent manner. *Neuron* 35, 291-306. 10.1016/S0896-6273(02)00769-9 12160747

[DMM023762C106] Pospisilik J. A., Schramek D., Schnidar H., Cronin S. J. F., Nehme N. T., Zhang X., Knauf C., Cani P. D., Aumayr K., Todoric J. et al. (2010). Drosophila genome-wide obesity screen reveals hedgehog as a determinant of brown versus white adipose cell fate. *Cell* 140, 148-160. 10.1016/j.cell.2009.12.027 20074523

[DMM023762C107] Prokop A. and Meinertzhagen I. A. (2006). Development and structure of synaptic contacts in Drosophila. *Semin. Cell Dev. Biol.* 17, 20-30. 10.1016/j.semcdb.2005.11.010 16384719

[DMM023762C108] Qian L. and Bodmer R. (2012). Probing the polygenic basis of cardiomyopathies in Drosophila. *J. Cell. Mol. Med.* 16, 972-977. 10.1111/j.1582-4934.2012.01529.x 22268758 PMC3337951

[DMM023762C109] Rajan A. and Perrimon N. (2013). Of flies and men: insights on organismal metabolism from fruit flies. *BMC Biol.* 11, 38 10.1186/1741-7007-11-38 23587196 PMC3626883

[DMM023762C110] Rinella M. E. and Sanyal A. J. (2015). NAFLD in 2014: genetics, diagnostics and therapeutic advances in NAFLD. *Nat. Rev. Gastroenterol. Hepatol.* 12, 65-66. 10.1038/nrgastro.2014.232 25560844 PMC4984668

[DMM023762C111] Rodríguez Cruz P. M., Palace J. and Beeson D. (2014). Inherited disorders of the neuromuscular junction: an update. *J. Neurol.* 261, 2234-2243. 10.1007/s00415-014-7520-7 25305004

[DMM023762C112] Rosen D. R. (1993). Mutations in Cu/Zn superoxide dismutase gene are associated with familial amyotrophic lateral sclerosis. *Nature* 362, 59-62. 10.1038/364362c0 8446170

[DMM023762C113] Sandoval H., Yao C.-K., Chen K., Jaiswal M., Donti T., Lin Y. Q., Bayat V., Xiong B., Zhang K., David G. et al. (2014). Mitochondrial fusion but not fission regulates larval growth and synaptic development through steroid hormone production. *Elife* 3, e03558 10.7554/eLife.03558 PMC4215535 25313867

[DMM023762C114] Santalla M., Valverde C. A., Harnichar E., Lacunza E., Aguilar-Fuentes J., Mattiazzi A. and Ferrero P. (2014). Aging and CaMKII alter intracellular Ca2+ transients and heart rhythm in Drosophila melanogaster. *PLoS ONE* 9, e101871 10.1371/journal.pone.0101871 25003749 PMC4087024

[DMM023762C115] Schott J.-J., Benson D. W., Basson C. T., Pease W., Silberbach G. M., Moak J. P., Maron B. J., Seidman C. E. and Seidman J. G. (1998). Congenital heart disease caused by mutations in the transcription factor NKX2–5. *Science* 281, 108-111. 10.1126/science.281.5373.108 9651244

[DMM023762C116] Sherwood N. T., Sun Q., Xue M., Zhang B. and Zinn K. (2004). Drosophila spastin regulates synaptic microtubule networks and is required for normal motor function. *PLoS Biol.* 2, e429 10.1371/journal.pbio.0020429 15562320 PMC532392

[DMM023762C117] Simon J. C. and Dickinson M. H. (2010). A new chamber for studying the behavior of Drosophila. *PLoS ONE* 5, e8793 10.1371/journal.pone.0008793 20111707 PMC2811731

[DMM023762C118] Soares L., Parisi M. and Bonini N. M. (2014). Axon injury and regeneration in the adult Drosophila. *Sci. Rep.* 4, 6199 10.1038/srep06199 25160612 PMC4145289

[DMM023762C119] Stark W. S. and Wasserman G. S. (1972). Transient and receptor potentials in the electroretinogram of Drosophila. *Vision Res.* 12, 1771-1775. 10.1016/0042-6989(72)90049-1 4627901

[DMM023762C120] Storm T., Emma F., Verroust P. J., Hertz J. M., Nielsen R. and Christensen E. I. (2011). A patient with cubilin deficiency. *N. Engl. J. Med.* 364, 89-91. 10.1056/NEJMc1009804 21208123

[DMM023762C121] Stowers R. S. and Schwarz T. L. (1999). A genetic method for generating Drosophila eyes composed exclusively of mitotic clones of a single genotype. *Genetics* 152, 1631-1639. 10430588 10.1093/genetics/152.4.1631 PMC1460682

[DMM023762C122] Sturtevant A. H. (1959). Thomas Hunt Morgan. *Biogr. Mem. Natl. Acad. Sci.* 33, 283-325.

[DMM023762C123] Tanouye M. A. and Wyman R. J. (1980). Motor outputs of giant nerve fiber in Drosophila. *J. Neurophysiol.* 44, 405-421. 6774064 10.1152/jn.1980.44.2.405

[DMM023762C124] Venken K. J. T. and Bellen H. J. (2014). Chemical mutagens, transposons, and transgenes to interrogate gene function in Drosophila melanogaster. *Methods* 68, 15-28. 10.1016/j.ymeth.2014.02.025 24583113 PMC4061744

[DMM023762C125] Venken K. J. T., Simpson J. H. and Bellen H. J. (2011). Genetic manipulation of genes and cells in the nervous system of the fruit fly. *Neuron* 72, 202-230. 10.1016/j.neuron.2011.09.021 22017985 PMC3232021

[DMM023762C126] Wagner C., Isermann K., Fehrenbach H. and Roeder T. (2008). Molecular architecture of the fruit fly's airway epithelial immune system. *BMC Genomics* 9, 446 10.1186/1471-2164-9-446 18823557 PMC2566315

[DMM023762C127] Wahlstedt-Froberg V., Pettersson T., Aminoff M., Dugue B. and Grasbeck R. (2003). Proteinuria in cubilin-deficient patients with selective vitamin B12 malabsorption. *Pediatr. Nephrol.* 18, 417-421. 12687456 10.1007/s00467-003-1128-y

[DMM023762C128] Wang T. and Montell C. (2007). Phototransduction and retinal degeneration in Drosophila. *Pflugers Arch.* 454, 821-847. 10.1007/s00424-007-0251-1 17487503

[DMM023762C129] Wang J., Kean L., Yang J., Allan A. K., Davies S. A., Herzyk P. and Dow J. A. T. (2004). Function-informed transcriptome analysis of Drosophila renal tubule. *Genome Biol.* 5, R69 10.1186/gb-2004-5-9-r69 15345053 PMC522876

[DMM023762C130] Wangler M. F., Yamamoto S. and Bellen H. J. (2015). Fruit flies in biomedical research. *Genetics* 199, 639-653. 10.1534/genetics.114.171785 25624315 PMC4349060

[DMM023762C131] Wartiovaara J., Öfverstedt L.-G., Khoshnoodi J., Zhang J., Mäkelä E., Sandin S., Ruotsalainen V., Cheng R. H., Jalanko H., Skoglund U. et al. (2004). Nephrin strands contribute to a porous slit diaphragm scaffold as revealed by electron tomography. *J. Clin. Invest.* 114, 1475-1483. 10.1172/JCI22562 15545998 PMC525744

[DMM023762C132] Wasserthal L. T. (2007). Drosophila flies combine periodic heartbeat reversal with a circulation in the anterior body mediated by a newly discovered anterior pair of ostial valves and ‘venous’ channels. *J. Exp. Biol.* 210, 3707-3719. 10.1242/jeb.007864 17951411

[DMM023762C133] Watson M. R., Lagow R. D., Xu K., Zhang B. and Bonini N. M. (2008). A drosophila model for amyotrophic lateral sclerosis reveals motor neuron damage by human SOD1. *J. Biol. Chem.* 283, 24972-24981. 10.1074/jbc.M804817200 18596033 PMC2529125

[DMM023762C134] Weavers H., Prieto-Sánchez S., Grawe F., Garcia-López A., Artero R., Wilsch-Bräuninger M., Ruiz-Gómez M., Skaer H. and Denholm B. (2009). The insect nephrocyte is a podocyte-like cell with a filtration slit diaphragm. *Nature* 457, 322-326. 10.1038/nature07526 18971929 PMC2687078

[DMM023762C135] West R. J., Furmston R., Williams C. A. and Elliott C. J. (2015). Neurophysiology of Drosophila models of Parkinson's disease. *Parkinsons Dis.* 2015, 381281 10.1155/2015/381281 25960916 PMC4414211

[DMM023762C136] Wolf M. J. and Rockman H. A. (2011). Drosophila, genetic screens, and cardiac function. *Circ. Res.* 109, 794-806. 10.1161/CIRCRESAHA.111.244897 21921272 PMC3678974

[DMM023762C137] Wolf M. J., Amrein H., Izatt J. A., Choma M. A., Reedy M. C. and Rockman H. A. (2006). Drosophila as a model for the identification of genes causing adult human heart disease. *Proc. Natl. Acad. Sci. USA* 103, 1394-1399. 10.1073/pnas.0507359103 16432241 PMC1360529

[DMM023762C138] Wu C. F., Ganetzky B., Haugland F. N. and Liu A. X. (1983). Potassium currents in Drosophila: different components affected by mutations of two genes. *Science* 220, 1076-1078. 10.1126/science.6302847 6302847

[DMM023762C139] Xie H. B., Cammarato A., Rajasekaran N. S., Zhang H., Suggs J. A., Lin H.-C., Bernstein S. I., Benjamin I. J. and Golic K. G. (2013). The NADPH metabolic network regulates human alphaB-crystallin cardiomyopathy and reductive stress in Drosophila melanogaster. *PLoS Genet.* 9, e1003544 10.1371/journal.pgen.1003544 23818860 PMC3688542

[DMM023762C140] Yamagata K., Furuta H., Oda N., Kaisaki P. J., Menzel S., Cox N. J., Fajans S. S., Signorini S., Stoffel M. and Bell G. I. (1996). Mutations in the hepatocyte nuclear factor-4alpha gene in maturity-onset diabetes of the young (MODY1). *Nature* 384, 458-460. 10.1038/384458a0 8945471

[DMM023762C141] Yamamoto S., Jaiswal M., Charng W.-L., Gambin T., Karaca E., Mirzaa G., Wiszniewski W., Sandoval H., Haelterman N. A., Xiong B. et al. (2014). A drosophila genetic resource of mutants to study mechanisms underlying human genetic diseases. *Cell* 159, 200-214. 10.1016/j.cell.2014.09.002 25259927 PMC4298142

[DMM023762C142] Zechner R., Strauss J. G., Haemmerle G., Lass A. and Zimmermann R. (2005). Lipolysis: pathway under construction. *Curr. Opin. Lipidol.* 16, 333-340. 10.1097/01.mol.0000169354.20395.1c 15891395

[DMM023762C143] Zhang F., Zhao Y. and Han Z. (2013a). An in vivo functional analysis system for renal gene discovery in Drosophila pericardial nephrocytes. *J. Am. Soc. Nephrol.* 24, 191-197. 10.1681/ASN.2012080769 23291470 PMC3559487

[DMM023762C144] Zhang K., Li Z., Jaiswal M., Bayat V., Xiong B., Sandoval H., Charng W.-L., David G., Haueter C., Yamamoto S. et al. (2013b). The C8ORF38 homologue Sicily is a cytosolic chaperone for a mitochondrial complex I subunit. *J. Cell Biol.* 200, 807-820. 10.1083/jcb.201208033 23509070 PMC3601355

